# Impact of Fluoxetine on Herbivorous Zooplankton and Planktivorous Fish

**DOI:** 10.1002/etc.5525

**Published:** 2022-12-16

**Authors:** Malgorzata Grzesiuk, Eva Gryglewicz, Piotr Bentkowski, Joanna Pijanowska

**Affiliations:** ^1^ Department of Hydrobiology, Faculty of Biology University of Warsaw Warsaw Poland; ^2^ Department of Biochemistry and Microbiology, Institute of Biology Warsaw University of Life Sciences Warsaw Poland; ^3^ tier3 Solutions Leverkusen Germany; ^4^ Faculty of “Artes Liberales” University of Warsaw Warsaw Poland

**Keywords:** Antidepressants, pharmaceutical pollution, *Daphnia*, fish, prey–predator interactions

## Abstract

The contamination of freshwater environments by pharmaceuticals is a growing problem. Modern healthcare uses nearly 3000 substances, many of which are designed to work at low dosages and act on physiological systems that have been evolutionarily conserved across taxa. Because drugs affect the organisms from different trophic levels, pharmaceutical pollution is likely to disturb species interactions. However, such effects are still only poorly understood. We investigated the impacts of environmentally relevant concentrations of the common drug fluoxetine (Prozac), an increasingly common contaminant of European waters, on predation behavior of crucian carp (*Carassius carassius*), a common planktivorous European fish, and the somatic growth of its prey, the water flea (*Daphnia magna*), a widespread planktonic crustacean. We exposed these two organisms to environmentally relevant levels of fluoxetine (360 ng L^−1^): the fish for 4 weeks and the water fleas for two generations. We tested the growth of the daphnids and the hunting behavior (reaction distance at which fish attacked *Daphnia* and feeding rate) of the fish under drug contamination. We found that *Daphnia* exposed to fluoxetine grew larger than a nonexposed cohort. The hunting behavior of *C. carassius* was altered when they were exposed to the drug; the reaction distance was shorter, and the feeding rate was slower. These effects occurred regardless of *Daphnia* size and the treatment regime they were subjected to. Our results suggest that contamination of freshwater environments with fluoxetine can disrupt the top‐down ecological control of herbivores by reducing the hunting efficiency of fish and, as a consequence, may lead to increases in cladoceran population numbers. *Environ Toxicol Chem* 2023;42:385–392. © 2022 The Authors. *Environmental Toxicology and Chemistry* published by Wiley Periodicals LLC on behalf of SETAC.

## INTRODUCTION

Modern healthcare makes use of nearly 3000 pharmaceutical substances. Especially over the last few decades, the production, prescription, and use of pharmaceuticals have sharply increased (Bernhardt et al., 2017; Kookana et al., 2014). As a result, pharmaceutical products regularly enter and contaminate freshwater ecosystems worldwide (Hughes et al., 2013; Mole & Brooks, 2019) and are found in tissues of wild animals (Puckowski et al., 2016). Environmental concentrations of these chemicals are often low (in the range of ng L^−1^; Mole & Brooks, 2019), because pharmaceuticals are typically designed to exert their biological effects at low doses. Moreover, they usually act on physiological pathways that are evolutionarily conserved across taxa (Arnold et al., 2014), with the majority (65%–86%) of them shared between humans and fish (Brown et al., 2014). Thus, the presence of biologically active substances in freshwater ecosystems can disturb aquatic biota and requires more detailed research.

A pharmaceutical commonly prescribed as an antidepressant is fluoxetine (commercial name Prozac), a selective serotonin reuptake inhibitor (SSRI). Fluoxetine and its metabolites that humans excrete are only partially removed by the currently applied wastewater treatment processes (Kookana et al., 2014), and the substance undergoes minimal degradation or transformation in sewage over months (Redshaw, 2008). Among clinically used SSRIs, fluoxetine is the most frequently found in surface waters of Europe, in concentrations from 0.8 to 596 ng L^−1^ (see Hughes et al., 2013; Mole & Brooks, 2019).

Several studies have demonstrated the effects of fluoxetine on behaviors of various fish species, such as mating, feeding, predator avoidance, and aggression (Barry, 2013; Di Poi et al., 2013; Lynn et al., 2007; Thoré et al., 2020; Weinberger & Klaper, 2014). The gap in knowledge concerning the long‐term effects of environmentally relevant concentrations of fluoxetine on freshwater organisms from different trophic levels is slowly being filled (see Mole & Brooks, 2019). More studies have involved extended exposure periods, which represents more realistic scenarios approximating full life cycles for some species (Martin et al., 2019; Thoré et al., 2020; Thoré, Brendonck, et al., 2021; Thoré, Philippe, et al., 2021; Thoré, Van Hooreweghe, et al., 2021; Wiles et al., 2020).

Fish play a significant role within aquatic food webs, moving between trophic levels during their ontogeny. Many fish, when young, feed on herbivorous zooplankton, and the filter‐feeding cladocerans, *Daphnia* among them, constitute an essential food for planktivorous fish. The structure and composition of a zooplankton community translate, via bottom‐up control, into the characteristics of the ichthyofauna.

Trophic webs are complex systems and cannot be simply understood as the sum of their elements; thus studying how pharmaceuticals affect a particular species is merely a step toward understanding the interactions affected by these chemicals within an aquatic community. Direct effects of pharmaceuticals on organisms (i.e., at the species level) can lead to shifts in community composition and can impact the trophic structure of aquatic ecosystems. Grzesiuk, Spijkerman, et al. (2018) demonstrated that algae exposed to ibuprofen increased survival rate in *Daphnia* compared with untreated algae. There is a need to study how neuroactive drugs, fluoxetine among them, affect species' interactions and generate ecological risks.

Our study, as an attempt to fill the existing gap, examined the effects of environmentally relevant levels of fluoxetine on a planktivorous fish and a cladoceran *Daphnia*, representing a simple predator–prey relationship in freshwater ecosystems. Our general hypothesis was that fluoxetine in low, environmentally relevant concentrations (360 ng L^−1^) will interfere with the prey–predator interaction. The effects of fluoxetine on the feeding behavior of fish have been described in a few studies, for example, by Gaworecki and Klaine (2008), who showed that fish cultured with fluoxetine need more time to capture their prey, or Thoré et al. (2019) and Thoré et al., (Thoré, Brendonck, et al., 2021; Thoré, Philippe, et al., 2021; Thoré, Van Hooreweghe, et al., 2021), who reported that fish exposed to antidepressants need a longer time before attacking prey. Weinberger & Klaper (2014) observed a lower feeding rate of fish in the presence of fluoxetine, compared with nontreated fish. Fluoxetine can also affect the fitness of invertebrates, altering their behavior, body size, and reproduction (Campos et al., 2012; Nielsen & Roslev, 2018; Rivetti et al., 2016). This can also affect predator–prey relationships. To gain a more complete picture of the overall impact of pharmaceuticals in the wild, we examined the effects of fluoxetine on the organisms from two trophic levels separately, and at the same time, on their interaction.

We chose filter‐feeding *Daphnia* (as the primary consumer) and the planktivorous crucian carp (*Carassius carassius*; as the predator). As predators, most fish species depend on vision (Guthrie, 1986), and the probability of a successful attack of fish on its prey is proportional to the reaction distance (Confer & Blades, 1975), which is the measure of the distance between the fish and prey when the fish locates the prey's position (see Confer et al., 1978). The reaction distance depends on the physical conditions of aquatic environment (e.g., light and turbidity; Utne, 1997), prey characteristics (e.g., size, color, transparency, and motion; Holmes & McCormick, 2010), and predator size (i.e., Blaxter & Straines, 1970). Because reaction distance depends on prey size and fluoxetine reduces *Daphnia* size (Campos et al., 2012), pharmaceutical effects on both predator and prey can affect hunting efficiency. Our hypothesis predicts that environmentally relevant fluoxetine concentrations will decrease the fish reaction distance, thus disturbing feeding efficiency. We examined the effects of environmentally relevant levels of fluoxetine on a planktivorous fish and cladoceran *Daphnia*, representing a simple predator–prey relationship in freshwater ecosystems.

To evaluate the effect of the antidepressant on hunting efficiency, we cultured both *C. carassius* and *D. magna*, with and without fluoxetine. In addition to studying how life‐long exposure to fluoxetine influences *Daphnia's* body size, we conducted experiments to determine 1) fish reaction distance, and 2) fish feeding rate within a given observation time in relation to their exposure to fluoxetine.

## MATERIALS AND METHODS

### Tested species and holding conditions

We obtained 5–6‐cm long crucian carps (*C. carassius*) from the Inland Fisheries Institute in Żabieniec, Poland for use in all our experimental treatments. We first divided the fish into two groups of seven individuals each. We exposed the first group to a constant concentration of fluoxetine (360 ng L^−1^), with the second group serving as the untreated control. We kept fish from each group in a 40 × 25 × 25‐cm glass tank filled with 20 L of previously charcoal‐filtered water. The tanks were individually aerated. Water with or without pharmaceutical addition were exchanged once a week. Fish were fed ad libitum with live *D. magna* and/or frozen Chironomidae larvae each day.

We cultured a clonal mixture of *D. magna* from a Warsaw city pond in Książęca Park (1 individual/100 ml) for three generations to eliminate the intraclonal differentiation caused by the maternal effects. We used neonates from the second maternal clutch to establish each subsequent generation. We fed the *Daphnia* daily with a given amount of green algae *Acutodesmus obliquus* to maintain a concentration of 1 mg C_org_ L^−1^. We changed the water every second day to renew the conditions just described. Furthermore, we exposed half of the *Daphnia* to fluoxetine for two generations in the same concentration as the treatment group of fish.

We kept all the experimental animals under a 16:8‐h light:dark cycle and a temperature of 20 ± 1 °C. In the experimental tank, the mean luminosity was 0.26 µmol photons m^−2^ s^−1^ in the experimental tank and 0.65 µmol photons m^−2^ s^−1^ in the culture room.

In freshwater systems, *D. magna* generally do not coexist with fish because this species' large body size makes it an easy target for visual predators such as fish, leading to its elimination. However, *D. magna* has been used as a model organism in various studies, especially in ecotoxicological tests. Their ecophysiology differs only slightly from smaller *Daphnia* that do coexist with crucian carps (Canton & Adema, 1978; Seidendorf et al., 2010).

### Exposures

We purchased fluoxetine HCl (greater than 98%) from Sigma‐Aldrich. We provided daily inserts of defined amounts of pharmaceutical solution into the water to maintain a fluoxetine concentration of 360 ng L^−1^ in each fluoxetine treatment group, assuming that a concentration of 360 ng fluoxetine L^−1^ was realistic for surface water (maximum concentration found reached 596 ng L^−1^; Hughes et al., 2013; Mole & Brooks, 2019). Fluoxetine is subject to photodegradation; its half‐time of decay is 55.6 ± 2.3 h (Heimke & Hartter, 2000; Lam et al., 2005). Thus the amount of the agent lost due to photolysis during 24 h was taken into account.

We began the exposure of the fish to fluoxetine 4 weeks prior to the behavioral assays, and we exposed the *Daphnia* for at least two generations. The individuals from the first generation were transferred to fluoxetine treatments within 2 h from hatching, whereas the second generation were in exposure all their life prior to the fish‐feeding experiments.

### Prey size

As just mentioned, we cultured *D. magna* for at least two generations with and without fluoxetine. We measured daphnid length starting from the top of the head to the edge of the carapace with the tail spine excluded. Length of the smaller (5‐day‐old) and larger (7‐day‐old) *Daphnia* with eggs was measured using the program NIS (Nikon NIS Elements; Table 1).

**Table 1:** Average (mean ± SD) length of *Daphnia* individuals cultured in experimental conditions for at least two generations

Small 5‐day old individuals without eggs		Large 7‐day old individuals with eggs	
Control (*n* = 26)	Fluoxetine (*n* = 26)	Control (*n* = 26)	Fluoxetine (*n* = 26)
1.79 ± 0.14 mm	1.88 ± 0.07 mm	2.74 ± 0.13 mm	2.87 ± 0.12 mm

For the study on fish reaction distance and feeding rate, we used four groups (26 individuals each) of *Daphnia*: 1) small, 5‐day‐old individuals without eggs not exposed to fluoxetine (SDc); 2) small, 5‐day‐old individuals without eggs exposed to fluoxetine (SDf); 3) large 7‐day‐old individuals with eggs not exposed to fluoxetine (LDc); and 4) large 7‐day‐old individuals with eggs exposed to fluoxetine (LDf).

### Reaction distance

We conducted our experiments in the same room where the organisms were kept. In the pre‐experimental phase, during fish exposure to fluoxetine, we habituated both groups of fish to hunt in an elongated and narrow experimental tank (100 × 10 × 15 cm) filled with charcoal‐filtered, aerated water to the depth of 12.5 cm. After several repeats, which occurred at least once a week, the fish showed no behavioral signs of stress like fast swimming or erratic changes of direction of movement, and began to hunt almost immediately after being placed in the experimental tank. Fish were not fed for approximately 18 h prior to the experimental trials.

We constructed a rail parallel to the experimental tank to film the fish hunting behavior. We then moved a human‐operated camera (Canon EOS 5D Mark II) along the rail, following the fish as they hunted *Daphnia*. The parallel rail ensured filming at an exact distance and angle from the camera to the fish, which was crucial for determining their reaction distance.

We placed the fish individually into the left side of the experimental tank and held them there in the net until we introduced five *Daphnia*, all from one of the introduced treatments (trials), in the tank. Recording began after the fish were released from the net and allowed to swim free.

**Figure 1:**
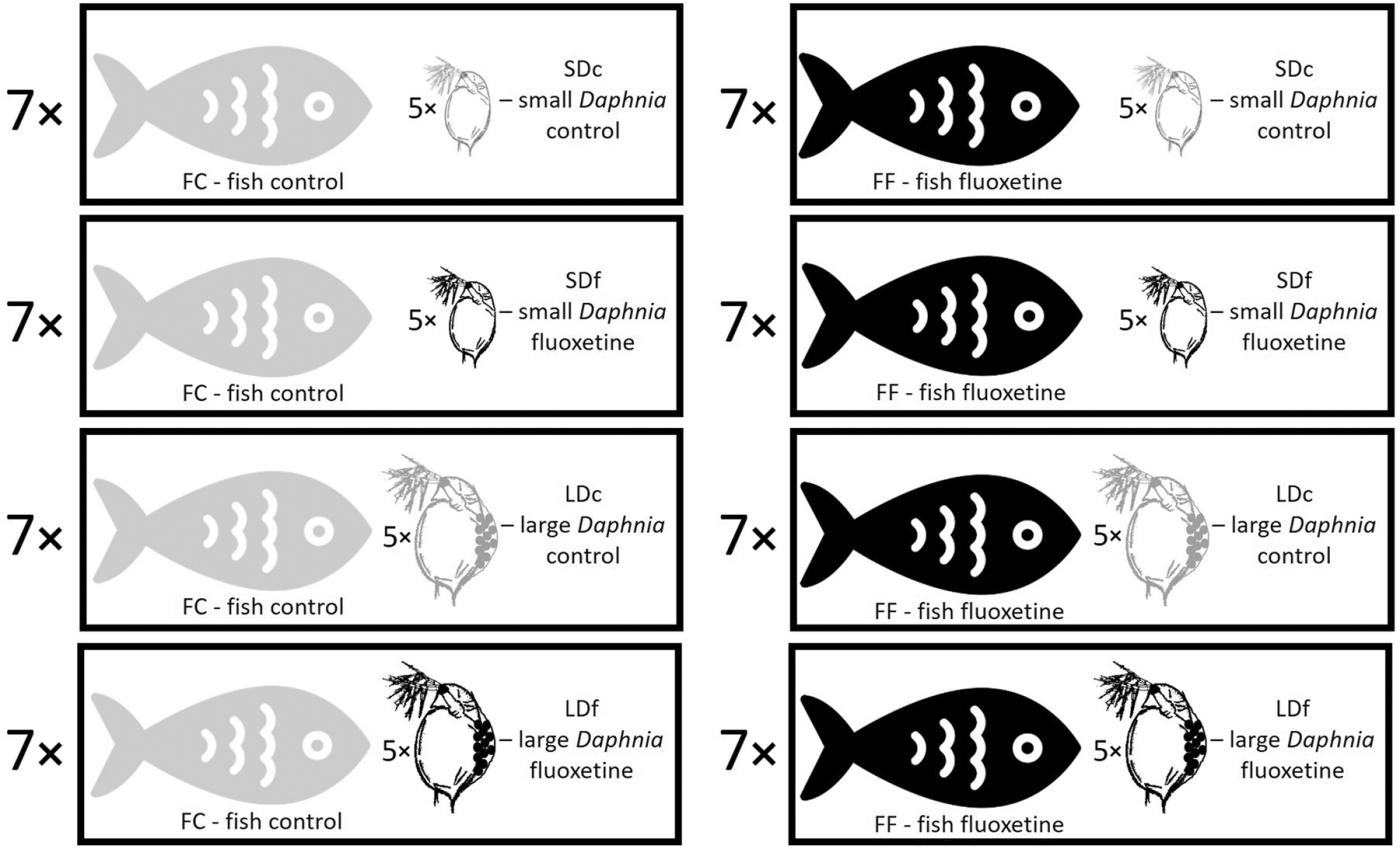
The experimental setting, which consisted of eight treatments. SDc = small *Daphnia* control; SDf = small *Daphnia* fluoxetine; LDc = large *Daphnia* control; LDf = large *Daphnia* fluoxetine.

The experimental setting consisted of eight treatments (Figure 1). One fish was observed at a time. Each fish hunted alone in the tank for 5 min, with its prey being individuals of one of the *Daphnia* groups. Each prey group was fed to the fish separately (Figure 1). Afterward, we removed each fish and counted the remaining *Daphnia* to assess the number of successful fish attacks to measure fish feeding efficiency. Finally, we analyzed the films and identified the exact moments when the fish spotted its prey just before it began to move toward it. Fish freezing for a split second before commencing an attack could indicate this moment. We defined the reaction distance as the maximum distance between the fish's eye and *Daphnia* at that second. We analyzed photoshots of these moments with the program Reaction Distance, which calculated this measure using calibration marks (manual marking of the fish eye and *Daphnia*). We analyzed the reaction distance of fish at the moment of first attack.

### Feeding rate

For measuring fish feeding rate, we used the same experimental setup as we did for testing reaction distance. In this series of tests, however, the experimental setting consisted of four groups: fish control were placed once in the experimental tank with: 1) five SDc (small *Daphnia* control) and five LDc (large *Daphnia* control); 2) five SDf (small *Daphnia* fluoxetine) and five LDf (large *Daphnia* fluoxetine): fish fluoxetine were placed once in the experimental tank with; 3) five SDc and 5 LDc; and 4) five SDf and five LDf. We allowed the fish to hunt for 3 min, and afterward we removed each fish from the tank and counted the remaining *Daphnia* individuals from both size classes.

### Statistical analysis

We performed all of the statistical analyses using Statistica 13 software. To check for the effect of fluoxetine on *Daphnia* size, we applied a two‐way analysis of variance (ANOVA) followed by a Tukey's honestly significant difference post hoc analysis. To check for effects of prey size, fish and prey treatment on fish reaction distance, and feeding efficiency, we performed a repeated‐measure three‐way ANOVA followed by a Tukey's honestly significant difference post hoc analysis.

## RESULTS

### Prey size

**Figure 2:**
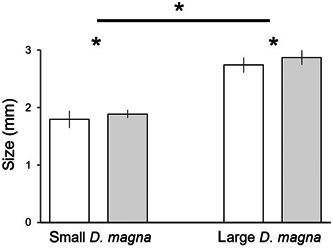
Size (mean ± SD) of small (5‐day‐old) and large (7‐day‐old) *Daphnia magna* individuals cultured without (white bars) and with fluoxetine (gray bars).

Although younger *Daphnia* individuals were significantly smaller than older ones (two‐way ANOVA *F*_(1,100)_ = 1583.4, *p* < 0.0001; Tables 1 and 2 and Figure 2), fluoxetine had a significant effect on *Daphnia* size in both age categories (Table 2): *Daphnia* cultured with fluoxetine were on average 90 and 130 µm larger than individuals cultured without the antidepressant, for 5‐ and 7‐day‐old individuals, respectively (Figure 2).

**Table 2:** Two‐way analysis of variance testing the effect of treatment (no fluoxetine, with fluoxetine) and age (5, 7 days) effect on *Daphnia* size

Variable	Factor	*df*	*F*	*p*‐value
*Daphnia* size				
	Treatment (*T*)	1	20	**<0.001**
	Age (*A*)	1	1583	**<0.001**
	*T* × *A*	1	1	0.448

Bold type indicates a significant value.

### Reaction distance

**Figure 3:**
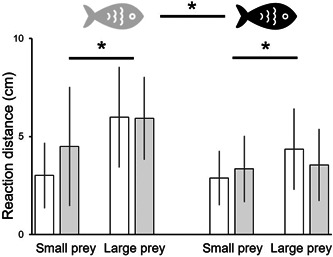
Reaction distance (mean ± SD) of crucian carps cultured without (gray fish symbol) and with fluoxetine (black fish) toward 5‐day‐old (small) and 7‐day‐old (large) *Daphnia magna* individuals cultured without (white bars) and with fluoxetine (gray bars). Asterisks with horizontal bar represent significant differences (*p* < 0.05) in fish reaction distance toward small and large prey (regardless of whether *Daphnia* were cultured with or without fluoxetine), and asterisks with horizontal bar between fish symbols represent significant differences (*p* < 0.05) in reaction distance of crucian carps cultured without and with fluoxetine (three‐way analysis of variance, post hoc Tukey honestly significant difference).

The reaction distance of the crucian carps toward their prey was affected by both prey size and fish treatment, but not by prey treatment. First, prey size significantly affected the fish reaction distance (Table 3). The fish reaction distance increased with the size of its prey (Figure 3). On average, the reaction distance toward 7‐day‐old *Daphnia* was greater by 1.55 cm than toward 5‐day‐old *Daphnia*. Second, fish treatment, that is, culturing with or without pharmaceuticals, significantly affected the fish's reaction distance (Table 3). On average, fish cultured with fluoxetine showed a reaction distance that was shorter by 5 cm in comparison with those cultured without the antidepressant, meaning that fish cultured with fluoxetine must be closer to the prey to launch an attack (Figure 3). Although fluoxetine affects prey size (Table 1; Figure 2), *Daphnia* culturing conditions did not affect the reaction distance of fish cultured with or without the pharmaceutical (Table 3).

**Table 3:** Repeated‐measure three‐way analysis of variance testing the effect of fish (predator) treatment (no fluoxetine, with fluoxetine), *Daphnia* (prey) size and *Daphnia* (prey) treatment (no fluoxetine, with fluoxetine) effect on fish reaction distance

Variable	Factor	Wilks value	*F*	Effect *df*	Error *df*	*p*‐value
Reaction distance						
	Fish treatment (*F*)	0.25	213.2	2	141	**<0.001**
	*Daphnia* size (*D*_s_)	0.89	8.7	2	141	**<0.001**
	*Daphnia* treatment (*D*_t_)	0.99	0.3	2	141	0.711
	*F* × *D*_s_	0.97	1.8	2	141	0.165
	*F* × *D*_t_	0.99	0.7	2	141	0.479
	*D*_s_ × *D*_t_	0.97	2.3	2	141	0.102
	*F* × *D*_s_ × *D*_t_	0.99	0.7	2	141	0.888

Bold type indicates significant value.

### Feeding rate

**Figure 4:**
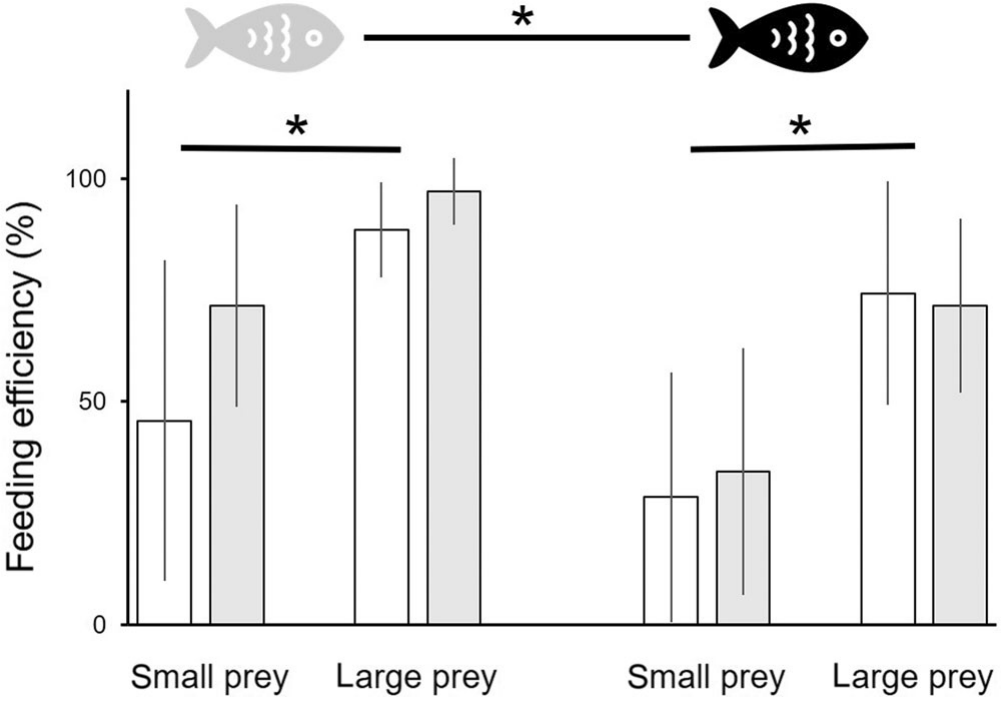
Feeding rate (mean ± SD; percentage of eaten prey) of crucian carps cultured without (gray fish symbol) and with fluoxetine (black fish). Prey were 5‐day‐old and 7‐day‐old *Daphnia magna* individuals cultured without (white bars) and with fluoxetine (gray bars). The fish were allowed to feed for 5 min. Asterisks with horizontal bar represent significant differences (*p* < 0.05) in feeding efficiency of fish toward small and large prey (regardless of whether *Daphnia* were cultured with or without fluoxetine), and asterisks with horizontal bar between fish symbols represent significant differences (*p* < 0.05) in feeding efficiency of crucian carps cultured without and with fluoxetine (three‐way analysis of variance, post hoc Tukey honestly significant difference).

Similar to the reaction distance, the feeding rate of the crucian carps was affected by both prey size and fish treatment, but not by the *Daphnia* treatment. Prey size significantly affected fish feeding rate (Table 4): fish ate almost twice as much large prey as small, 83% versus 44% of offered prey (Figure 4). Moreover, the feeding rate depended on whether the fish had been exposed to fluoxetine (Table 4). Fish not exposed to fluoxetine ate on average 20% more prey within a 5‐min time frame than exposed fish (Figure 4).

**Table 4:** Repeated‐measure three‐way analysis of variance testing the effect of fish (predator) treatment (no fluoxetine, with fluoxetine), *Daphnia* (prey) size and *Daphnia* (prey) treatment (no fluoxetine, with fluoxetine) effect on fish feeding rate

Variable	Factor	Wilks value	*F*	Effect *df*	Error *df*	*p*‐value
Feeding rate						
	Fish treatment (*F*)	0.95	6.4	2	261	**<0.01**
	*Daphnia* size (*D*_s_)	0.77	39.1	2	261	**<0.001**
	*Daphnia* treatment (*D*_t_)	0.99	0.2	2	261	0.773
	*F* × *D*_s_	1	0.06	2	261	0.943
	*F* × *D*_t_	0.99	0.1	2	261	0.892
	*D*_s_ × *D*_t_	1	0.03	2	261	0.971
	*F* × *D*_s_ × *D*_t_	0.99	0.2	2	261	0.817

Bold type indicates significant value.

## DISCUSSION

Very low concentrations of pharmaceuticals, as registered in freshwater ecosystems, have recently been shown to affect organisms from different trophic levels: 1) primary producers such as algae (see, Grzesiuk, Spijkerman, et al., 2018); 2) primary consumers such as *Daphnia* (see, Grzesiuk, Mielecki, et al., 2018; Nielsen & Roslev, 2018); and 3) first and higher level predators (see Thoré, Brendonck, et al., 2021; Thoré, Van Hooreweghe, et al., 2021; Thoré, Philippe, et al., 2021; Thoré et al., 2019). The results of our study demonstrated that exposure to fluoxetine caused an increase in *Daphnia* body size and reduced fish feeding efficiency. We also showed that the interaction of predator and prey is affected in a fluoxetine‐contaminated environment by reducing the predator's feeding rate and decreasing fish responsiveness to the presence of prey.

### Prey size

We have shown for the first time that *Daphnia* cultured for two generations with environmentally relevant concentrations of fluoxetine are significantly larger than those not exposed (Figure 2). This result is in opposition to data shown by Campos et al. (2012) who, however, used higher fluoxetine concentrations (1000, 4000, and 8000 ng L^−1^) than those most commonly found in the environment (Mole & Brooks, 2019). Campos et al. (2012) also found that *Daphnia* exposed to SSRIs, for example, fluoxetine, changed their perception of food conditions, switching their life‐history to the pattern typical for food‐rich environments, that is, producing offspring earlier and in greater numbers, even in food‐depleted conditions. This happens at the expense of neonates' smaller size and individual's increased vulnerability to low oxygen levels. In our experiments, we cultured *Daphnia* in nonlimiting levels of oxygen and food.

Our results may be explained by the mode of action of fluoxetine. Injection of fluoxetine increased glucose and hyperglycemic hormonal levels in hemolymph of several species of decapods (Fanjul‐Moles, 2006). Hyperglycemic hormone, with several others produced and released by the X–organ–sinus gland complex is known to regulate reproduction, nutrient metabolism, chromatic adaptation, and growth (Webster et al., 2012).

Moreover, the increased *Daphnia* body size could be a positive effect of low concentrations of toxic substances (hormesis). A study on how fluoxetine in a wide range of concentrations, from 10^−1^ to 10^5^ ng L^−1^, alters phototactic behavior of *D. magna* showed a nonmonotonic effect across this range. Negative phototactic behavior of adult females decreased from 1 to 10^3^ ng L^−1^ of fluoxetine, that is, within the range of concentrations detected in freshwaters, whereas no effect was observed at higher concentrations (Rivetti et al., 2016). A nonmonotonic effect on reproduction was shown for other neuroactive drugs: carbamazepine, diazepam, and propranolol in a wide range of concentrations (Rivetti et al., 2016). These, and many other results support the postulate of using environmentally realistic concentrations of pharmaceutical in experimental ecological studies.

### Reaction distance and feeding rate

As mentioned, fluoxetine affects different kinds of fish behavior such as mating, feeding, predator avoidance, and aggression (Barry, 2013; Di Poi et al., 2013; Lynn et al., 2007; Weinberger & Klaper, 2014). Our study is the first to demonstrate the effect of environmentally‐relevant concentrations of fluoxetine on fish reaction distance to prey. Both reaction distance toward *Daphnia* and feeding rate significantly decreased when fish were exposed to fluoxetine (Figure 3). Similarly to observations made by Brodin et al. (2013), we found that fish exposed to antidepressants ate significantly fewer prey in comparison with untreated animals (Figure 4).

Vision‐driven fish predation on *Daphnia* is size dependent, with larger prey favored (Brooks & Dodson, 1965; Zaret, 1980). The results from our study demonstrate that larger *Daphnia* were easier for fish to spot (i.e., the reaction distance was greater for larger *Daphnia*), which further supports the earlier published data (Gliwicz et al., 2010; Werner & Hall, 1974). The average reaction distance toward 7‐day‐old *Daphnia* was greater by 1.55 cm than toward 5‐day‐old *Daphnia*. Even though *Daphnia* cultured with fluoxetine were statistically larger on Days 5 and 7 (Figure 2) than animals cultured without the antidepressant, the fish reaction distance did not depend on the prey culturing conditions. Moreover, it is known that fluoxetine alters *Daphnia* phototactic behavior. Simão et al. (2019) reported a decreased response to light in *Daphnia*, which had been exposed to the antidepressant: 1) individuals moved less; 2) stayed closer to the bottom; and 3) stayed closer together at low light intensities. One might expect that fluoxetine's effect on *Daphnia* (larger size, decreased mobility) would work in favor of fish predation; the reaction distance toward these individuals did not, however, differ significantly from the reaction to unexposed *Daphnia*.

Consistent with previous studies on fish, we observed an apparent overall decline of interest in hunting in treated fish. For example hybrid striped bass (*Morone saxatilis* × *M. chrysops*) exhibited a concentration‐ and exposure duration‐dependent decrease in the ability to capture prey (Gaworecki & Klaine, 2008). Brain serotonin activity of bass was measured, showing that increased time to capture prey correlated with decreases in brain serotonin activity (Gaworecki & Klaine, 2008). However, these experiments were conducted at higher fluoxetine concentrations and with shorter exposure of animals than applied in our experiments. It is postulated that fluoxetine can act as an appetite suppressor (De Pedro et al., 1998). In this case, fish motivation to seek food may not be high, resulting in a low reaction distance. Neuroactive drugs such as fluoxetine should be added to the list of factors, next to predator size, prey characteristics (color, transparency, and motion), and physical conditions of the aquatic environment (light and turbidity) influencing reaction distance.

### Possible consequences for the food web

We have demonstrated the following impact of fluoxetine on animals from two trophic levels and the predator–prey relationship between these species: 1) *Daphnia* grew larger in the presence of fluoxetine (Figure 2); and 2) fish ate less prey in the environment contaminated by antidepressants, (Figure 4). To conclude, fluoxetine affected *Daphnia* size in both age categories, thus making them more vulnerable to predation. However, this effect was countered by decreased threat from planktivorous fish due to their feeding behavior, which was impaired by fluoxetine. Larger size of *Daphnia* in the presence of fluoxetine may result in a higher filtration rate (see Burns, 1969) and thus more efficient control of algal biomass. At the same time, fish influenced by fluoxetine fed less efficiently and,thus,the biomass of herbivorous zooplankton was less effectively controlled. These two phenomena can theoretically favor filter‐feeding herbivores, eventually leading to more efficient top‐down control of algae.

## Supporting Information

The Supporting Information is available on the Wiley Online Library at https://doi.org/10.1002/etc.5525.

## Conflict of Interest

The authors declare no conflict of interest.

## Author Contributions Statement

**Malgorzata Grzesiuk:** Conceptualization; Data curation; Formal analysis; Funding acquisition; Investigation; Methodology; Project administration; Resources; Supervision; Validation; Visualization; Writing—original draft; Writing—review & editing. **Eva Gryglewicz:** Investigation; Methodology; Writing—original draft; Writing—review & editing. **Piotr Bentkowski:** Methodology; Software; Writing—review & editing. **Joanna Pijanowska:** Conceptualization; Funding acquisition; Investigation; Methodology; Project administration; Resources; Writing—review & editing.

## Supporting information

This article includes online‐only Supporting Information.

raw_data.

## Data Availability

Raw data can be found in the Supporting Information.
